# How Community Pharmacists Perceive Ethics in Clinical Research: A Qualitative Study

**DOI:** 10.3390/healthcare9111496

**Published:** 2021-11-02

**Authors:** Miku Ogura, Rieko Takehira, Tatsuya Watanabe, Etsuko Arita

**Affiliations:** 1 Laboratory of Medical Psychology, Pharmaceutical Education Research Center, Kitasato University School of Pharmacy, Tokyo 108-8641, Japan; dp18203@st.kitasato-u.ac.jp; 2 Kitasato Clinical Research Center, Kitasato University School of Medicine, Sagamihara 252-0374, Japan; tatuya-w@insti.kitasato-u.ac.jp

**Keywords:** clinical research, ethics, community pharmacists, qualitative research, research ethics education

## Abstract

In recent years, the importance of building evidence in clinical practice that is increasingly acknowledged globally has been recognized in Japan as well, and it is expected that clinical research by community pharmacists will grow. In Japan, however, community pharmacists have few opportunities to learn about research ethics and may lack the training to make ethical decisions. We conducted a questionnaire survey of community pharmacists (*n* = 200) using a free descriptive format to understand how they perceived research ethics. Our qualitative analysis of 170 respondents revealed various perspectives (<A pharmacist’s grounding>, <How pharmacists perceive research>, and <Ethical issues entailed by research>) of Japanese pharmacists on ethics in the context of clinical research. With respect to how to understand research, the following perspectives were found: “research that prioritizes researchers,” “research that prioritizes research subjects (patients),” and “research that enters into regular work.” The perspectives on “research that prioritizes research subjects (patients)” and “research that enters into regular work” may inadvertently lead to ethically inappropriate research due to mismatch in professional values or poor understanding of research. These findings can contribute to the development of an educational program for community pharmacists on research ethics.

## 1. Introduction

In Japan, the focus of pharmacist work has shifted from solely dispensing drugs to also include communication with patients. To fulfill their societal mission of providing a people-focused service, pharmacists must consider not only the needs of patients that require immediate attention but also the needs of future patients and the medical system as a whole. In recent years, the importance of contributing to medical care by building evidence in clinical practice has been recognized in Japan, and further research by pharmacists is required. As such, pharmacists will also gain increasing opportunities to engage in clinical research aimed at addressing problems in clinical settings.

All clinical research in Japan must comply with the relevant laws and ethical standards [1,2]. These rules also include ethical considerations for participants. Researchers must have the ability to make appropriate ethical decisions. However, Japanese community pharmacists have limited knowledge of and few opportunities to learn about research ethics, suggesting that they may lack the training to make the ethical decisions to suit the clinical research in question [3]. Currently, attempts are underway to establish educational interventions aimed at improving ethical decision-making [4]. The emergence of ethical passivity shows the complexity of ethical decision-making [5]. Therefore, it is important to understand the perception of research ethics, including the general ethical passivity of community pharmacists; this makes it possible to build an educational program that suits their actual situation. The authors of this study have been developing a learner-driven educational program that links knowledge of research ethics to practice. As part of this project, we conducted an opinion survey among community pharmacists.

The purpose of this study was to develop descriptive survey data concepts that describe how community pharmacists perceive research ethics, with the ultimate aim of contributing to the development of an educational program on research ethics.

## 2. Materials and Methods

### 2.1. Survey Method

Between 20 February and 6 March 2018, an anonymous online questionnaire survey was conducted. This survey was targeted at community pharmacists and was outsourced to an academic research company (medical marketing promotion research). Community pharmacists who registered with the company as monitors were invited to participate in this survey through a dedicated website. There was one attempt to request them to participate. In addition, this survey was an exploratory study. Therefore, the target number of samples was set to 200, and the survey was completed when 200 respondents were obtained. The questionnaire surveyed the respondents’ demographic background (gender, age, years of work, promotion of clinical research at their workplace, opportunities to consider ethical issues at their workplace, etc.). Further, using a free descriptive format, respondents described how they perceived ethics in the context of clinical research. Of the responses obtained, textual data from 170 respondents were analyzed. The data for 30 people was excluded from the analysis because the answers were not suitable for analysis (responses such as “Nothing in particular”).

### 2.2. Analytical Method

The data were analyzed qualitatively in four steps. In Step 1 (segmentation), descriptive textual data were segmented. In Step 2 (codification), each segment was assigned a code representing the context to which the segment belonged. In Step 3 (categorization), codes that resemble each other were grouped together into categories. In Step 4 (abstraction), the categories were distributed on a spatial network to illustrate how the categories relate to each other. The codification was undertaken using a qualitative data analysis tool called Steps for Coding and Theorization (SCAT) [6,7]. Categorization and abstraction were undertaken using the scheme proposed by Jiro Kawakita in his KJ-Method [8,9]. At each step, the members of the research team conferred on the results. When differences of opinion arose, the members discussed the differences until they reached a consensus. All members reviewed the final analysis results. This rigorous analytical process enhanced the reliability and validity of the results.

### 2.3. Ethical Considerations

The Kitasato Institute Hospital Research Ethics Committee determined that the study protocol required no ethical review before the study commenced. No personal identifying data were collected, and the survey did not involve highly invasive content.

## 3. Results

### 3.1. Basic Data

Of the 170 respondents from whom textual data were obtained, 61 were men and 109 were women. Eight were aged 20–29, 67 were aged 30–39, 49 were aged 40–49, 37 were aged 50–59, and nine were aged 60 or over. The average work experience for a pharmacist was 16.1 (SD = 8.5) years. Thirteen were promoting clinical research at their workplace, and nine had the opportunity to consider ethical issues at their workplace.

### 3.2. Qualitative Analysis of Descriptive Statements

**Figure 1:**
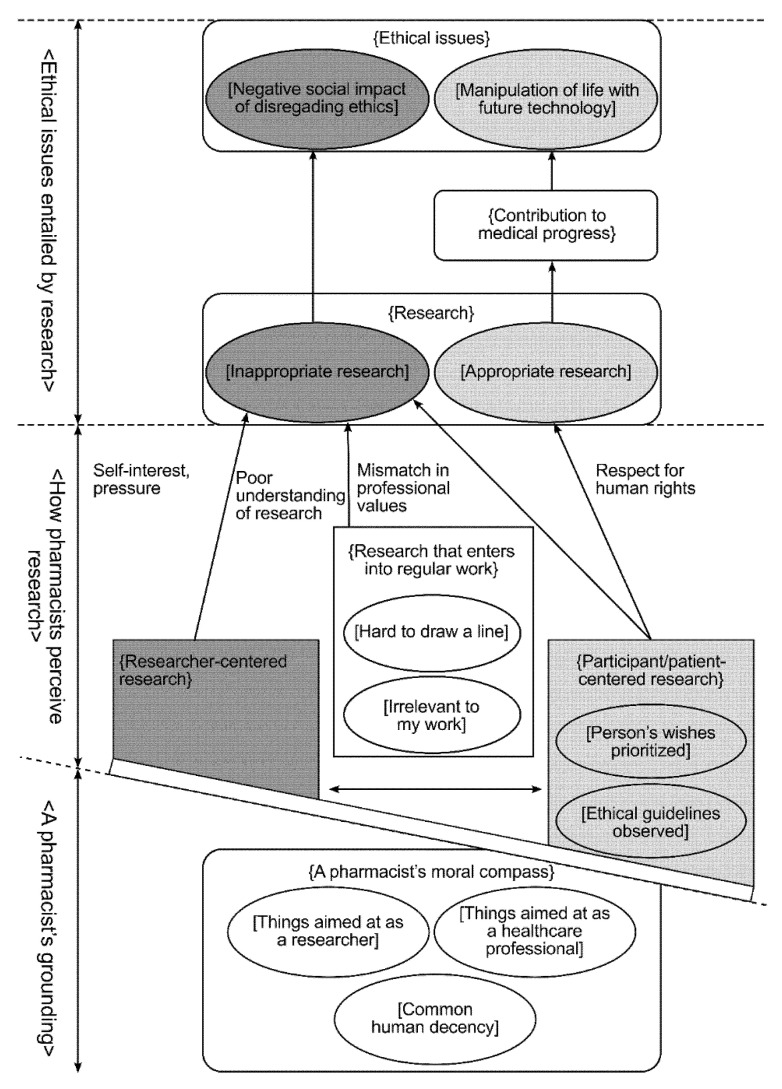
Conceptual diagram of pharmacists’ thoughts on ethics. Note: <> = Categories; {} = Subcategories; [] = Subsubcategories.

The qualitative analysis of the descriptive responses about ethics in clinical research yielded seven subcategories and 11 sub of subcategories, which this paper refers to as subsubcategories. Table 1 shows the categories, subcategories and subsubcategories, along with a sample code representing a typical code for the subsubcategory in question. Figure 1 shows the spatial network illustrating the relationships between the categories.

In the following sections, categories are identified by angle brackets <>; subcategories are identified by curly brackets {}; subsubcategories are identified by square brackets [].

### 3.3. Description of Spatial Network

Three broad categories were identified in the spatial network. We labeled the first category <A pharmacist’s grounding> because its subcategories describe the underlying values that community pharmacists associate with research ethics. The second category was labeled <How pharmacists perceive research> because its subcategories describe how community pharmacists feel about a research project they are about to engage in. The third category was labeled <Ethical issues entailed by research> because this category describes ethical issues encountered when conducting research.

The spatial network was arranged to reflect all the relationships between the three categories. <A pharmacist’s grounding> is positioned at the bottom. The category includes the subcategory {A pharmacist’s moral compass}, which, itself, consists of three subsubcategories. Of the other two categories, <How pharmacists perceive research> is positioned in the middle, while <Ethical issues entailed by research> is at the top. <How pharmacists perceive research> has three subcategories: {Researcher-centered research}, {Participant/patient-centered research}, and {Research that enters into regular work}. All three subcategories had a pathway to {Research}, which is one of the subcategories in <Ethical issues entailed by research>. Beyond {Research} lies {Ethical issues}, the second of the two subcategories in this category.

#### 3.3.1. A Pharmacist’s Grounding

Community pharmacists’ ethical values are underpinned by common human decency. They are also informed by their professional training and career, as described in the subsubcategories [Things aimed at as a researcher] and [Things aimed at as a healthcare professional]. These three components join together to constitute a pharmacist’s moral compass.

#### 3.3.2. How Pharmacists Perceive Research

Pharmacists perceive research in a variety of ways depending on their knowledge of research ethics and the way they relate their work to research. Their perspectives were categorized into the following three subcategories: researcher-centered research, participant/patient-centered research, and research that enters into regular work. The first two subcategories were diametrically opposed. {Participant/patient-centered research} consists of the following subsubcategories: [Person’s wishes prioritized] and [Ethical guidelines observed]. {Research that enters into regular work} describes the feeling that it is [hard to draw a line] between research and healthcare work and that research is [Irrelevant to my work].

#### 3.3.3. Ethical Issues Entailed by Research

All three subcategories of <How pharmacists perceive research> are linked, in accordance with their respective characteristics, to the {Research} subcategory in <Ethical issues entailed by research>. {Researcher-centered research} led to [Inappropriate research] in that the researchers prioritized their own interests or succumbed to outside pressure. Conversely, {Participant/patient-centered research} led to [Appropriate research] in that the researchers felt obliged to safeguard research participants and patients, out of their respect for human rights. However, the same subcategory also had a pathway to [Inappropriate research], where there was a mismatch in professional values. The third subcategory, {Research that enters into regular work}, was linked to [Inappropriate research], in that the researcher poorly understood the research.

{Research} consists of subsubcategories [Inappropriate research] and [Appropriate research]. Both inappropriate and appropriate research led to respective subsubcategories of ethical issues. [Inappropriate research] led directly to the subsubcategory [Negative social impact of disregarding ethics], while [Appropriate research] led indirectly, via the subcategory {Contribution to medical progress}, to the subsubcategory [Manipulation of life with future technology].

## 4. Discussion

In this study, we conducted a qualitative analysis to induce concepts that describe how community pharmacists perceive ethics when embarking on clinical research.

Of the three categories yielded in the analysis, we first discuss <A pharmacist’s grounding>. From this category, we extracted {A pharmacist’s moral compass}, which consisted of three subsubcategories. Of these subsubcategories, [Common human decency] suggests that community pharmacists derive their ethical worldview from moral values they have acquired in Japanese moral education and social life. However, the other two subsubcategories, [Things aimed at as a researcher] and [Things aimed at as a healthcare professional], suggest that their ethical worldview is also informed by professional values. {A pharmacist’s moral compass}, therefore, implies that pharmacists base their decisions and actions both on the interests of the patient and on the interests of furthering medical science. Miklich et al. reported that professionally engaged pharmacists “think and behave in ways that positively affect patients’ health and advance the profession’s values and societal mission” [10] (p. 5). Additionally, medical actions are considered sound if they conform to the four principles of biomedical ethics (autonomy, beneficence, nonmaleficence, and justice) [11]. Therefore, the professional values [Things aimed at as a researcher] and [Things aimed at as a healthcare professional], are both necessary components of a pharmacist’s moral compass.

Regarding the second categories, <How pharmacists perceive research>, the subcategories {Researcher-centered research} and {Participant/patient-centered research} indicate what the pharmacist prioritizes when engaging in the research. The other subcategory, {Research that enters into regular work}, implies that there is no clear demarcation between clinical research and regular pharmaceutical work. Our analysis revealed that researcher-centered research results can lead to inappropriate research: when pharmacists place excessive weight on researcher interests, they are more likely to manipulate or distort the results in their own favor or in favor of a corporate sponsor. This concern gained attention in Japan following the Diovan scandal, in which it was revealed that the clinical trial data for the drug Diovan had been doctored to exaggerate its efficacy [12]. The impact of this scandal made community pharmacists aware that research misconduct can occur in clinical research.

{Participant/patient-centered research} consists of two subsubcategories. The first subsubcategory, [Person’s wishes prioritized], conforms to one of the four principles of biomedical ethics: autonomy [11]. The other, [Ethical guidelines observed], indicates an intention to observe laws and standards pertaining to medical research. These subsubcategories imply that pharmacists are mindful of ethical validity when they place top priority on the interests of the participants or patients. However, the analysis also revealed that {Participant/patient-centered research} can still lead to inappropriate research. {Participant/patient-centered research} leads to [Appropriate research], provided that the pharmacist gives sufficient weight to respecting participant/patient autonomy and observing ethical guidelines, so as to safeguard human rights and thus ensure ethical validity. However, if there is a mismatch in professional values, they risk committing (Inappropriate research). “Professional values” refer to the values that a researcher or pharmacist has acquired through work. Researchers and pharmacists must understand that the purpose of research differs from that of medical practice: the purpose of medical research is to advance medical science by obtaining findings that are valuable to society, while the goal of medical practice is to cure disease by providing the best possible healthcare to the patient in front of the practitioner. The relationship between the patient and healthcare professional that exists in healthcare settings shifts in research settings into a relationship between the participants and the researcher. When pharmacists engage in research, they assume the role of the researcher (as opposed to the pharmacist) in the researcher–participant relationship. Such pharmacists risk deviating from the research protocol if they, despite assuming this role, focus only on meeting the needs of the patient, as described in the subsubcategory [Person’s wishes prioritized]. Thus, even if the pharmacist commits to {Participant/patient-centered research}, they may inadvertently commit [Inappropriate research] if they fail to differentiate between the professional values of a researcher and those of a healthcare professional.

As for the third subcategory in this category, {Research that enters into regular work}, like the other two, this perception leads to [Inappropriate research]. The reason for this is the subcategory’s two subsubcategories: [Hard to draw a line] and [Irrelevant to my work]. Both subsubcategories suggest a poor understanding of research. The scientific process prevails in clinical research. A number of studies have polled pharmacists on their views about clinical research and reported that pharmacists struggle with research in general because they lack an understanding of how to conduct it [13,14,15]. If pharmacists have a poor grasp of research in general, this may explain the subsubcategories. Specifically, pharmacists may find it difficult to draw a line because they lack confidence in conducting research, and they may find research [Irrelevant to my work] because of their lack of familiarity with it. According to one report, pharmacists are interested in clinical research because they want to solve problems faced by the patients they encounter in their daily work [16]. If pharmacists engage in research out of a desire to resolve a clinical question, it is essential that they understand the ethical considerations for participants. In summary, the perception of research as something that {Research that enters into regular work}, with its subsubcategories [Hard to draw a line] and [Irrelevant to my work], reveals that the researcher can inadvertently commit [Inappropriate research] if the researcher lacks the scientific and ethical awareness necessary for research. Our analysis revealed that [Inappropriate research] can result not only from researcher-centered research that prioritizes the interests of researchers, but also from other factors such as a lack of research knowledge or a mismatch in professional values. To avoid engaging in inappropriate research practices, community pharmacists must master the ability to cope with ethical issues based on an understanding of the laws and standards pertaining to research ethics [1,2] and an awareness of past examples of research malpractice [17,18]. In a concept analysis of medical professionals’ ethical sensitivity, Aoyagi identified three abilities that such professionals require in medical practice: the ability to detect ethical issues, the ability to clarify them, and the ability to deal with them [19]. Thus, for pharmacists working in clinical settings to gain ethical literacy, they must first understand what research entails. They must then familiarize themselves with research ethics and how to comply with them and develop their ability to deal with ethical issues. This kind of learning gives pharmacists a deeper understanding of research ethics and the ability to respond to ethical issues so that they can conduct research more appropriately.

Finally, we discuss the subcategories in <Ethical issues entailed by research>. Two of these subcategories are research and ethical issues. {Research} consists of subsubcategories [inappropriate research] and [Appropriate research]. [Inappropriate research] links to the subsubcategory (Negative social impact of disregarding ethics), denoting the pharmacists’ perception that research malpractice constitutes a violation of social norms. [Appropriate research] links to the subcategory {Contribution to medical progress}, suggesting that they see clinical research as a way to contribute to society; {Contribution to medical progress}, in turn, links to the subsubcategory (Manipulation of life with future technology), implying that progress in medical science is accompanied by ethical issues. The two subsubcategories of ethical issues are the negative social impact of disregarding ethics and the manipulation of life with future technology. The first subsubcategory, insofar as it is connected with research malpractice, denotes the issue as an ethical issue in relation to the fairness of research. As for [Manipulation of life with future technology], this subsubcategory denotes that applying future technology involves issues related to life and death, and thus constitutes an issue for biomedical ethics. Thus, community pharmacists perceive {Ethical issues} from two viewpoints: that of research malpractice and of biomedical ethics.

### Limitations

There may be some possible limitations in this study. First, the respondents were only those who registered as monitors with an academic research company and who responded to the invitation to participate in the study. Generalization is limited because it does not reflect the opinions of those who have not registered as monitors and those who are registered but did not participate in the survey. Second, since this survey used a free descriptive format, we were unable to determine the reasons behind some participants not sharing their description of ethics. Third, our study did not consider the thinking processes that pharmacists undergo during the research. Future studies should aim to ascertain the thinking processes pharmacists exhibit as they shift between their roles as researchers and healthcare professionals. Such research could provide insights to guide the development of an ethics educational program which prepares pharmacists to cope with the ethical issues they will face.

## 5. Conclusions

Our qualitative analysis revealed diverse perspectives (<A pharmacist’s grounding>, <How pharmacists perceive research>, and <Ethical issues entailed by research>) on ethics in the context of clinical research. The results suggest that, depending on how one perceives ethics, the process may inadvertently lead to ethically inappropriate research.

## Figures and Tables

**Table 1:** Category, Subcategories, Subsubcategories, and Sample of Codes.

Categories	Subcategories	Subsubcategories	Sample of Codes
a pharmacist’s grounding	a pharmacist’s moral compass	common human decency	Never deviate from common sense
		things aimed at as a researcher	Never get too caught up in my own interests
		things aimed at as a healthcare professional	A pharmacist should act in a circumspective manner
how pharmacists perceive research	researcher-centered research		Cozy relationship with business
	participant/patient-centered research	person’s wishes prioritized	Prioritizing the wishes of patients
		ethical guidelines observed	First priority is to safeguard the interests of participants
	research that enters into regular work	hard to draw a line	It is difficult to draw a line as to how far it is permissible to do with participants
		irrelevant to my work	No need for it in everyday work
ethical issues entailed by research	research	inappropriate research	Doctoring data
		appropriate research	Ensuring the reasonableness and validity of the research
	contribution to medical progress		Hope to help advance medical science in ethical way
	ethical issues	negative social impact of disregarding ethics	Need to rethink approach taking into consideration past scandal over hypertension drug
		manipulation of life with future technology	Cloning technology

Note: Subsubcategories = sub of subcategories.

## Data Availability

Data are available on request.

## References

[1] Ministry of Health, Labour and Welfare Clinical Trials Act. https://www.mhlw.go.jp/file/06-Seisakujouhou-10800000-Iseikyoku/0000213334.pdf.

[2] Baba S., Toda I. (2019). Ethics-related legal amendments and ethics applications for medical research. J. Jpn. Soc. Oral Implant..

[3] Arita E., Ogura M., Takehira R. (2021). Research ethics education for community pharmacists—a survey for developing a program to motivate community pharmacists to learn about research ethics. Jpn. J. Pharm. Educ..

[4] Yamane Y., Unzai H., Inada Y., Kadoya S. (2019). Review of research ethics education in Japan. Jpn. Soc. Sci. Educ..

[5] Cooper R.J., Bissell P., Wingfield J. (2008). Ethical decision-making, passivity and pharmacy. J. Med. Ethics.

[6] Yahata S., Takeshima T., Kenzaka T., Okayama M. (2021). Fostering student motivation towards community healthcare: A qualitative study. BMJ. Open.

[7] Miyahara S. (2020). Outcomes and problems of the librarian’s licensure examination in the Philippines: An analysis using steps for coding and theorization (SCAT). J. Educ. Libr. Inf. Sci..

[8] Taniguchi C., Okada A., Seto N., Shimizu Y. (2020). How visiting nurses detect symptoms of disease progression in patients with chronic heart failure. Int. J. Qual. Stud. Health Well-Being.

[9] Kono K., Goto Y., Hatanaka J., Yoshikawa E. (2017). Competencies required for occupational health nurses. J. Occup. Health.

[10] Miklich M.A., Reed B.N., Mattingly T.J., Haines S.T. (2016). Beliefs and behaviors of professionally engaged pharmacists. J. Am. Pharm. Assoc..

[11] Beauchamp T.L., Childress J.F. (2019). Principles of Biomedical Ethics.

[12] Nagai Y. (2019). History and future direction of academia clinical trial regulation: Lessons of Diovan affairs and concerns on clinical trials act. Jpn. J. Pharmacoepidemiol..

[13] Ishizuka N., Terahara F., Matsuki Y., Sakurada K., Nakamura Y., Suzuki C., Obara H., Kohara Y. (2020). Questionnaire survey on career formation, qualifications acquisition, and research activities of hospital pharmacists at Sapporo-Kosei general hospital. J. Jpn. Assoc. Rural Med..

[14] Ikemura M., Ando M., Hashida T. (2017). Proposal for an approach to support clinical research by pharmacists. Jpn. J. Pharm. Educ..

[15] Sawada Y., Takehira R., Yamamura S. (2015). Survey of attitude towards pharmacy practice research of pharmacists who work in drug stores. Bull. Grad. Sch. Josai. Int. Univ..

[16] Watanabe K., Yokoyama Y., Sato K., Takegami M., Sekine Y., Amioka K., Onishi Y., Fukuoka S. (2010). Educational analysis of levels of interest in clinical research among clinical pharmacists. Jpn. J. Pharrn. Health Care Sci..

[17] Beecher H.K. (1966). Ethics and clinical research. N. Engl. J. Med..

[18] Park J. (2017). Historical origins of the Tuskegee experiment: The dilemma of public health in the United States. Korean J. Med. Hist..

[19] Aoyagi Y. (2016). Concept analysis of medical professionals’ ethical sensitivity. J. Jpn. Acad. Nurs. Sci..

